# A Treatise on the Structure, Functions, and Diseases of the Foot and Leg of the Horse; Comprising the Comparative Anatomy of These Parts in Other Animals, &c.

**Published:** 1841-01

**Authors:** 


Art. IX.—*A Treatise on the Structure, Functions, and Diseases of the Foot and Leg of the Horse; comprising the Comparative Anatomy of these parts in other animals, &c.* By W. C. Spooner, m.r.v.c.—*London*, 1840. 8vo, pp. 337.

This is a very valuable work, and merits the attention not merely of such members of our body—a vast majority—as are directly interested in the practical part of the subject of which it treats, but also of those who pursue the study of comparative anatomy and comparative pathology, as objects of scientific enquiry. The author has evidently studied his profession in the proper manner.

